# In high responding patients undergoing an initial IVF cycle, elevated estradiol on the day of hCG has no effect on live birth rate

**DOI:** 10.1186/1477-7827-12-119

**Published:** 2014-11-28

**Authors:** Michael T Zavy, LaTasha B Craig, Robert A Wild, Sana N Kahn, Dena O’Leary, Karl R Hansen

**Affiliations:** Section of Reproductive Endocrinology and Infertility Department of Obstetrics and Gynecology, Oklahoma University Health Sciences Center, Oklahoma City, Oklahoma USA; Section of Uro-gynecology, Department of Obstetrics and Gynecology, Oklahoma University Health Sciences Center, Oklahoma City, Oklahoma USA

**Keywords:** IVF, Estradiol level, High responder, Live birth rate, Age, Embryo quality, Day of embryo transfer

## Abstract

**Background:**

The impact of elevated estradiol on the day of human chorionic gonadotropin (hCG) administration on in vitro fertilization (IVF) outcomes has been debated for over 25 years. Some investigators have shown a positive effect, others a negative effect; while most have shown no effect. Few studies have expressed their findings based on live birth. This study examined the relationship between estradiol level and other IVF cycle response parameters in relation to pregnancy, with a focus on live births after controlling for embryo quality.

**Methods:**

We performed a retrospective cohort study on 489 patients <40 years old that underwent an initial IVF cycle. Estradiol concentration on the day of hCG was categorized as; low <2000 pg/ml), mid (2001-4000 pg/ml) and high (>4000 pg/ml) to determine how estradiol level on the day of hCG affected response variables during the IVF cycle. We performed a subgroup analysis restricted to patients with good/fair quality embryos transferred (n = 428), to control for embryo quality and assessed pregnancy outcome. The association between estradiol and live birth (LB) was then evaluated after identifying and controlling for confounding factors. Multivariate analysis was used to identify significant main effects and interactions in the model. Estradiol levels were also compared in patients having a LB or not (NLB) in both populations.

**Results:**

We found that estradiol was significantly related to + hCG, clinical pregnancy rate, age, and most other IVF cycle response variables. After performing the subgroup analysis controlling for embryo quality, we found that LB rates were not different. Only the main effects of average embryo quality at transfer (AEQS), age and transferring two embryos influenced LB. Estradiol levels were also compared in patients having a LB or NLB in both populations and was found to be higher/not different in LB patients. LB rates and AEQS were also not different in a subgroup of patients having an elevated level of estradiol (>4200 pg/ml) on the day of hCG in patients having embryo transfer on day 3 or day 5.

**Conclusions:**

After controlling for embryo quality, elevated estradiol on the day of hCG had no effect on LB.

## Background

In IVF cycles, high responding patients are characterized as having a large number of follicles that are associated with elevated levels of estradiol and frequently by elevated levels of progesterone on the day of hCG administration. In most situations, this results in an increased number of embryos from which candidates can be chosen for embryo transfer. Due to its potential clinical importance the impact of elevated estradiol on the day of hCG on IVF outcomes has been debated for over 25 years and even after this length of the time the issue of how, or if, the supra-physiological hormonal environment exhibited in high responding patients has an adverse effect on pregnancy outcome is still not settled. Regarding elevated estradiol and pregnancy outcome, some investigators [1–4] have shown a positive effect and others [5–8] a negative effect; while most studies [9–18] have shown no effect. A meta-analysis conducted in 2004 [19] reviewed nine studies and concluded that no high quality evidence existed to support or refute the value of estradiol levels on the day of hCG administration as a determining factor in establishing pregnancy in IVF cycles. Possibly contributing to the disparity in outcomes from these studies are differences in the way trials were conducted, including the use of repeated measures, patient exclusion criteria, type of stimulation, day of transfer, number of embryos transferred and how estradiol levels were categorized (cutoff, percentile or area under the curve). There is also considerable variation among studies in how pregnancy rates were expressed with most using clinical pregnancy rate, with or without implantation rate. Few studies have based their conclusions on live birth outcome which arguably provides the most information on the embryos ability to tolerate the hormone environment to which it has been exposed.

The purpose of this study was to determine the relationship between the levels of estradiol on the day of hCG administration and pregnancy outcomes, with emphasis on live birth rate after controlling for embryo quality. We addressed this by initially categorizing patients according to the level of estradiol on the day of hCG administration, which has been the basis of the majority of previously published studies. We then performed additional analyses controlling for embryo quality and for confounding and interactions occurring between IVF response variables. In a secondary analysis we compared differences in estradiol levels between patients achieving a live birth and those that did not (NLB), since differences in estradiol levels have been reported in previously published reports when stratified by birth outcome [17, 20, 21]. We also tested if the day of embryo transfer made a difference in live birth (LB) rate in patients with an elevated level of estradiol on the day of hCG since some prior studies [17, 20, 22] had suggested that elevated estradiol only had an adverse effect on pregnancy outcome when embryo transfer was conducted on day 3.

## Methods

### Study design

We performed a retrospective cohort analysis (2006-2012) on patients that underwent an initial IVF cycle at the OU Reproductive Medicine Clinic at the University of Oklahoma. Of the 500 patients evaluated, 489 patients were included in the present study. Eleven patients were excluded due to; failed fertilization (4), only abnormal embryos available for transfer (3) or all embryos cryopreserved due to threatened ovarian hyper-stimulation (4). This investigation was approved by the Oklahoma University Health Sciences Center Institutional Review Board (#2781, approved, 3/12/2013).

### Patient population and stimulation protocols

Patients < 40 years of age with any etiology of infertility were included in the study; no patients using donor eggs were included. Following a baseline ultrasound, all patients underwent stimulation with recombinant follicle-stimulating hormone (FSH; Follistim, Organon USA or Gonal-F, Sereno) and in the majority of cases, in combination with human menopausal gonadotropin (hMG; Menopur or Repronex, Ferring Pharmaceuticals). Total units of FSH administered were calculated for each patient with the exception of 19 patients in which this information was missing. Three different protocols were utilized for patients undergoing IVF during the study interval, including long gonadotropin releasing hormone (GnRH) agonist, micro-dose flare and GnRH antagonist protocols. The particular protocol chosen was based on anticipated patient response, with the majority of patients undergoing the long agonist protocol. Follicular monitoring was carried out via serial vaginal ultrasound and serum measurement of 17β-estradiol (estradiol) and adjustments made in gonadotropin dose based upon follicular development. When 2 or more follicles were ≥18 mm, patients received a subcutaneous injection of hCG (5000 or 10000 IU) and follicular aspiration took place 35-36 hours later. A blood sample was taken at the last ultrasound, prior to hCG administration and analyzed for estradiol. Luteal phase supplementation was by daily IM injection of progesterone (50 mg). Fifteen days following follicle aspiration, serum hCG was measured and considered positive if >20 mIU, biochemical pregnancy (BCP). Clinical pregnancy (CP) was documented by observing a fetal sac(s) and heart rate by vaginal ultrasound at 6.5 - 7 weeks.

### Hormone measurements

Serum levels of estradiol and hCG were measured by chemiluminescent enzyme immunoassays (Immulite; Diagnostic Products, Los Angeles, CA, USA). Inter-assay and intra-assay coefficients of variation were 9.8% and 9.4% for estradiol and 5.6% and 3.0% for hCG.

### IVF-embryo transfer

Following follicle aspiration, oocytes were held in modified human tubal fluid (mHTF) + 5% (volume/volume) human serum albumen (HSA) (In Vitro Care; IVC) until placed into individual drops of HTF + 5% HSA with oil overlay (Sage) in a humidified atmosphere of 6% CO_2_ and 94% air maintained at 37° until intracellular sperm injection (ICSI) or conventional IVF was carried out 4 to 6 hours following oocyte retrieval. Most cases (70%) utilized ICSI with 25% undergoing ICSI and conventional IVF (split) and (5%) undergoing conventional IVF only. After injection, oocytes were placed into 50ul micro-drops of IVC-1 + 5% HSA (In Vitro Care) or Global medium +10% global protein supplement (GPS; Life Global) using oil overlay and cultured in a humidified tri-gas atmosphere (5-6.5% CO_2_; 5% O_2_ and residual nitrogen) at 37°C. Fertilization was checked between 17 and 18 hours and normally fertilized oocytes having 2 pronuclei were placed in groups of 3-4 and cultured as described previously. On day 2, embryos were graded and embryos allocated to new culture drops based on quality (good, fair or poor). A decision was made to perform embryo transfer on day 3 or day 5, based on the number of good quality embryos. Blastocyst transfer was elected if there were at least 6 good/fair quality embryos. If this criterion was not met, there were a low number of embryos or poor quality embryos; transfer on day 3 was elected. New culture plates were made daily and embryo development and video documentation was also conducted daily at approximately 24 hour intervals.

### Embryo and blastocyst quality score at transfer

Cleavage stage embryos and blastocysts were graded as being good (1), fair (2) or poor quality (3) and numerical conversion of embryo grades were performed in order to statistically analyze the data. The embryos or blastocysts that were transferred for each patient were averaged to yield an average embryo quality score (AEQS). The numeric scoring is similar to that used by the Society for Assisted Reproductive Technology [23]. For cleavage stage embryos, the quality was determined by stage of development, blastomere symmetry and fragmentation of the embryos. Our grading system uses the following designation on day 3; A (good), B (fair) C (poor) with a (+/-) modifier for B quality embryos only. A good quality embryo (score of 1.0) had an A or B+ quality at the 6 cell to10 cell stage of development. A fair quality embryo (score of 2.0 for a “B” or 2.5 for a “B-”) at the 5 cell to 14 cell stage of development and a poor quality (score of 3) if the embryo was developmentally delayed (≤ 4 cells), had excessive fragmentation >40%) or extreme blastomere asymmetry. Blastocysts were graded as follows: a good quality blastocyst (score of 1) on day 5; the inner cell mass (ICM) quality was not less than B+ quality while the trophectoderm (TE) quality was no lower than B quality. In the case of blastocysts (+/-) modifiers were used for B quality for both the ICM and TE. The minimum stage of development of good quality blastocysts was 3 (expanded) or in the case of an early observation, 2 (cavitated). A day 6 good quality blastocyst was similar to day 5 except with more advanced development. A fair quality blastocyst (score of 2) for day 5; the minimum quality was 2BB- and for day 6 was 4BB-. A poor quality blastocyst (score of 3) for day 5 or day 6, included any blastocyst having a B-B, or lower quality regardless of stage of development.

### Response variables

We evaluated if the estradiol level on the day of hCG was associated with BCP, CP and LB, in patients undergoing their initial IVF cycle. Estradiol concentration on the day of hCG was categorized as; low <2000 pg/ml), mid (2001-4000 pg/ml) and high (>4000 pg/ml) to determine how estradiol level on the day of hCG affected response variables during the IVF cycle. Given that a higher estradiol level on the day of hCG may be related to better embryo quality, we controlled for this factor by evaluating the relationship between estradiol level and outcomes in a subpopulation (n = 428) containing only patients with good or fair embryo quality score at transfer. The poor quality group had an average embryo quality score range of 2.3 – 3.0. To focus on LB rates, we examined if there was an association between maternal estradiol levels and LB after controlling for potential confounders using multivariate logistic regression. We examined if estradiol levels and other response variables were different by live birth status. The NLB group included those patients that did not become pregnant, or had a BCP or a CP but not a LB. We examined what effect the day of embryo transfer had on NLB and LB rate in a subgroup of patients from the entire population having an estradiol level of >3000 pg/ml on the day of hCG. We compared LB rate, number of embryos transferred and embryo transfer quality scores in patients having embryo transfer on day 3 or day 5.

### Statistical analyses

We evaluated differences between estradiol groups for response variables using Chi-square and analysis of variance (ANOVA), considering p < 0.05 as significant. Chi-Square, Student’s t-tests and univariate logistic regression was used to evaluate the association between the live births and each covariate. Multivariable logistic regression was performed, including all covariates with a significance level of p ≤ 0.1 on univariable analysis, avoiding co-linearity. The model was assessed for the presence of two way interactions and confounding, with a difference between crude and adjusted odds ratios of >15% considered evidence of a significant confound in the regression model.

The entire population as well as the subgroup were partitioned by NLB or LB, and differences in response variables was assessed by two sample t-tests, with p < 0.05 considered significant. The same approach was taken when examining NLB and LB differences in the high estradiol populations by day of embryo transfer (day 3 or day 5). Chi square was used to evaluate differences in LB between days of transfer with a level of P < 0.05 considered significant.

## Results

Patient demographics for the entire population are shown in Table 1. Pregnancy rates and IVF cycle response parameters for the entire population (n = 489) and the sub-group (n = 428) that had only good or fair quality embryos transferred, are summarized in Tables 2 and 3 respectively. For the entire population (Table 2), the higher estradiol groups were associated with greater biochemical and clinical pregnancy rates. Whereas, in the sub-group (Table 3), with fair to good embryo quality no association with estradiol was observed on BCP, CPR or LBR in contrast to all other IVF response variables. Although live birth rates were greatest (NS, P = 0.052) in higher estradiol groups from the categorical analysis shown in Table 2, following the univariate analysis (Table 4) in which estradiol level was evaluated as a continuous variable, estradiol level had a significant (P = 0.013) effect on LB as did other covariates including age, FSH dosage, number of metaphase II (MII) oocytes, total 2PN embryos, AEQS and embryo transfer day. In the multivariate analysis (Table 5) however, estradiol level was not related to live birth. Only the main effects of AEQS, patient age, and the transfer of two embryos as opposed to 1 or 3, were found to significantly affect LB in the final model. The multivariate regression model also revealed significant two-way interactions between transfer on day 5 and patient age (P < 0.01) and transfer on day 5 and FSH dose administered (P < 0.04). IVF response variables in the total population and in the subgroup containing only good and fair quality embryos on the day of embryo transfer were evaluated by live birth status. In the total population for day 3 transfers (Table 6) there were differences between the NLB and LB groups in patient age, FSH dose, number of 2PN embryos and AEQS, whereas, in the subgroup-only analysis, patient age and AEQS were different. In the total population for day 5 transfers (Table 7), FSH dose, number of MII eggs and AEQS differed. In the subgroup however, only FSH dose differed between NLB and LB. In the entire population the LB rate was greater for day 5 than for day 3 transfers. However, when the subgroup containing only good and fair embryos was evaluated, although the LB rate was slightly higher (65.3% vs 59.1%), the difference was no longer statistically significant.

**Table 1:** **Patient demographics of the entire population (n = 489)**

Parameters	Values
Age (years)	31.6 ± 4.1
BMI	25.9 ± 6.2
FSH administered (mIU) per patient	2969 ± 1434
Estradiol on day of hCG (pg/ml)	2871 ± 1153
Oocytes retrieved per patient	18.9 ± 8.6
MII oocytes per patient	14.5 ± 7.2
2PN embryos per patient	9.8 ± 5.2
Embryos transferred per patient	2.04 ± 0.42
Embryo quality at transfer (AEQS) per patient	1.61 ± 0.59
Percentage of day 3 transfers	286 (58.5%)
Percentage of day 5 transfers	203 (41.5%)

Mean ± 1SD.

**Table 2:** **Estradiol groups in the entire population of patients and IVF outcome**

Variables	Group 1 < 2000 pg/mL, n = 123)	Group 2 (2000-4000) pg/ml, n = 288)	Group 3 > 4000 pg/mL, n = 78)	p- value
Age (years)	32.6 ± 4.2	31.5 ± 4.1	30.6 ± 3.8	<0.003
BMI	27.4 ± 7.0	26.3 ± 6.2	24.9 ± 6.1	<0.024
FSH dose (IU)*	3851 ± 1454	2883 ± 1314	1900 ± 896	<0.001
Peak estradiol (pg/mL)	1489 ± 365	2950.1 ± 561	4758 ± 594	<0.001
Oocytes retrieved	11.9 ± 5.7	19.9 ± 7.5	26.5 ± 8.6	<0.001
MII oocytes	8.9 ± 4.7	15.1 ± 6.3	20.7 ± 7.3	<0.001
Total 2PN	6.4 ± 3.7	10.3 ± 4.7	13.5 ± 5.9	<0.001
AEQS	1.78 ± 0.67	1.57 ± 0.58	1.48 ± 0.44	<0.001
No. embryos transferred	2.11 ± 0.50	2.04 ± 0.40	1.92 ± 0.35	<0.008
BCP (%)	58.5^a^	70.8^b^	78.2^b^	<0.007
CP (%)	49.6^a^	64.2^b^	65.4^b^	<0.015
LB (%)	47.2	59.0	61.5	0.052
Total pregnancy loss (%)	19.4	16.7	21.3	0.670
Poor quality embryos for transfer (%)	19.5	9.7	1.3	<0.001

Mean ± 1SD.

Chi-square and ANOVA tests as appropriate. For pregnancy related variables, within row, response variables with different superscripts differ at a minimum of P < 0.05. The number patients in the entire population were 489. *The n for FSH dose was 470 due to missing values.

**Table 3:** **Estradiol groups in a subpopulation having good or fair quality embryos for transfer and IVF outcome**

Variables	Group 1 (<2000 pg/mL, n = 95)	Group 2 (2000-4000 pg/mL, n = 257)	Group 3 > 4000 pg/mL, n = 76)	p- value
Age (years)	32.6 ± 4.1	31.4 ± 3.9	30.6 ± 3.9	<0.003
BMI	28.3 ± 7.1	26.2 ± 6.1	24.8 ± 6.1	<0.002
FSH dose (IU)*	3677 ± 1416	2834 ± 1311	1904 ± 901	<0.001
Peak estradiol (pg/mL)	1506 ± 352	2965 ± 564	4763 ± 599	<0.001
Oocytes retrieved	12.5 ± 5.6	20.2 ± 7.4	26.7 ± 8.4	<0.001
MII oocytes	9.6 ± 4.7	15.5 ± 6.3	20.9 ± 7.3	<0.001
Total 2PN	7.0 ± 3.5	10.6 ± 4.7	13.7 ± 5.8	<0.001
AEQS	1.49 ± 0.43	1.43 ± 0.43	1.46 ± 0.42	<0.490
No. embryos transferred	2.11 ± 0.45	2.00 ± 0.35	1.91 ± 0.33	<0.002
BCP (%)	71.6	74.3	78.9	<0.640
CP (%)	62.1	68.5	65.8	<0.550
LB (%)	58.9	63.0	61.8	<0.810
Total pregnancy loss (%)	17.6	15.2	21.7	<0.540

Mean ± 1SD.

Chi-square and ANOVA tests as appropriate. For pregnancy related variables, within row, response variables with different superscripts differ at a minimum of P < 0.05. The number patients in the subpopulation were 428. *The n for FSH dose for the subpopulation was 413 due to missing values.

**Table 4:** **Univariate analysis of the association of response variables with LB outcome**

	Live birth			
	No	Yes		p value
Age	32.26 ± 4.30	31.17 ± 3.85		0.001
Estradiol level on day of hCG	2724 ± 1158	2984 ± 1138		0.013
FSH (IU administered)	3305 ± 1462	2716 ± 1362		0.001
MII oocytes	13.14 ± 6.92	15.49 ± 7.27		0.001
Total 2PN	8.83 ± 5.13	10.61 ± 5.15		0.001
AEQS	1.820 ± 0.662	1.448 ± 0.476		0.001
Number of embryos transferred	2.075 ± 0.499	2.014 ± 0.351		0.100
Embryo transfer day			n	0.013
Day 3	138 (48.3%)	148 (51.7%)	286	
Day 5	75 (36.9%)	128 (63.1%)	203	
Total	213	276	489

**Table 5:** **Multivariate logistic regression**

Variables	Beta	Odds ratio	Confidence limit	p value
AEQS	-1.07832	0.34017	(0.23316 - 0.49628)	0.001
Patient age	-0.16244	0.85007	(0.73570 - 0.98221)	0.028
Estradiol level	-0.00011	0.99989	(0.99968 - 1.00010)	0.308
Day 5 Embryo transfer	-3.36729	0.03448	(0.00098 - 1.21223)	0.064
FSH dosage	-0.00089	0.99911	(0.99782 - 1.00040)	0.175
Two embryos transferred	1.05610	2.87512	(1.25510 – 6.58623)	0.013
Three embryos transferred	1.02113	2.77634	(0.91250 – 8.44714)	0.072
Day 5 transfer X Age	0.14946	1.16121	(1.03430 – 1.30369)	0.011
Day 5 transfer X FSH dosage	-0.00034	0.99966	(0.99933 – 0.99999)	0.042
Age X FSH dosage	0.00002	1.00002	(0.99999 – 1.00006)	0.224

**Table 6:** **Response variables for the entire population and subgroup having good/fair quality embryos transferred on day 3**

	Entire population		Good/fair embryos only	
Variables	Non-LB	LB	Non-LB	LB
Number	138 (48.3%)	148 (51.7%)*	96 (40.9%)	139 (59.1%)**
Patient age	33.0 ± 4.2^a^	31.4 ± 4.0^a^	32.9 ± 3.9^d^	31.5 ± 3.9^d^
FSH dose (IU)	3574 ± 1499^b^	3158 ± 1397^b^	3423 ± 1490	3124 ± 1381
Estradiol on day of hCG pg/ml	2451 ± 1082	2634 ± 1061	2588 ± 1115	2636 ± 1080
BMI	26.5 ± 6.6	27.1 ± 6.7	27.1 ± 6.4	27.2 ± 6.7
Number retrieved oocytes	15.9 ± 8.2	16.0 ± 6.9	17.2 ± 8.6	16.1 ± 6.8
MII eggs	11.1 ± 6.0	12.1 ± 5.7	12.0 ± 6.2	12.3 ± 5.6
Number of 2PN	6.9 ± 4.1^c^	8.1 ± 3.9^c^	7.8 ± 4.1	8.1 ± 3.8
Number of embryos transferred	2.19 ± 0.51	2.11 ± 0.29	2.17 ± 0.43	2.11 ± 0.32
AEQS	1.91 ± 0.68^a^	1.39 ± 0.49^a^	1.54 ± 0.42^a^	1.32 ± 0.40^a^

All values are Mean ± 1SD.

Differences within row comparing the same response variable by pregnancy status (NLB or LB) for the entire population or patients having only good or fair quality embryos transferred having the same superscript are different; ^a^p <0.001; ^b^p <0.020; ^c^p <0.010, ^d^p <0.007.

*Chi Square comparison for difference between Day 3 and Day 5 in the total population for Live Birth was p <0.02.

**Chi Square comparison for difference between Day 3 and Day 5 in population with only good or fair quality embryos used for transfer for Live Birth was NS.

**Table 7:** **Response variables for the entire population and subgroup having good/fair quality embryos transferred on day 5**

Variables	Entire population		Good/fair embryos only	
	Non-LB	LB	Non-LB	LB
Number	75 (36.9%)	128 (63.1%)*	67 (34.7%)	126 (65.3%)**
Patient age	30.9 ± 4.1	30.9 ± 3.7	30.6 ± 4.1	30.9 ± 3.7
FSH dose (IU)	2798 ± 1250^a^	2204 ± 1124^a^	2702 ± 1189^c^	2203 ± 1130^c^
Estradiol on day of hCG pg/ml	3226 ± 1132	3390 ± 1092	3375 ± 1088	3391 ± 1090
BMI	26.0 ± 6.7	25.5 ± 5.9	25.9 ± 6.7	25.4 ± 5.9
Number retrieved oocytes	21.6 ± 8.3	23.9 ± 8.3	22.4 ± 7.9	23.8 ± 8.3
MII eggs	16.9 ± 6.9^b^	19.4 ± 7.0^b^	17.7 ± 6.6	19.4 ± 7.0
Number of 2PN	12.2 ± 5.3	13.5 ± 4.9	12.7 ± 5.1	13.5 ± 4.9
Number of embryos transferred	1.87 ± 0.41	1.91 ± 0.36	1.85 ± 0.40	1.90 ± 0.37
AEQS	1.66 ± 0.60^d^	1.51 ± 0.46^d^	1.51 ± 0.42	1.49 ± 0.43

All values are Mean ± 1SD.

Differences within row comparing the same response variable by pregnancy status (NLB or LB) for the entire population or patients having only good or fair quality embryos transferred having the same superscript are different; ^a^p <0.001; ^b^p < 0.010, ^c^p < 0.007, ^d^p <0.040.

*Chi Square comparison for difference between Day 3 and Day 5 in the total population for Live Birth was p < 0.02.

**Chi Square comparison for difference between Day 3 and Day 5 in populations with only good or fair quality embryos used for transfer was NS.

In the final analysis, a subgroup of patients having embryo transfer on day 3 or day 5 that had an estradiol level of > 3000 pg/ml on the day of hCG were compared (Table 8). When embryo transfer was conducted on day 3 or day 5 with high quality embryos, patients had similar estradiol levels, with the number of oocytes retrieved, mature oocytes and number of 2PN embryos all being higher in the day 5 transfer group. However, there was no significant difference in the CPR or LBR for patients receiving a transfer on day 3 or day 5.

**Table 8:** Comparison between patients having transfer on day 3 or day 5 with estradiol levels >3000 pg/ml on the day of hCG with *high quality embryos transferred

Variables	Day 3	Day 5	p value
Number	52	80	
Estradiol level	3859 ± 595	4037 ± 799	0.17 NS
Oocytes collected	21.6 ± 8.4	26.4 ± 8.8	< 0.002
Number of MII oocytes	15.2 ± 6.4	21.7 ± 7.2	< 0.0001
Number of 2PN embryos	8.5 ± 4.2	15.2 ± 5.5	< 0.0001
Number of embryos transferred	2.04 ± 0.19	1.85 ± 0.36	< 0.006
AEQS	1.18 ± 0.24	1.23 ± 0.25	0.25 NS
CPR	33/52 (63.5%)	55/80 (68.7%)	NS
LBR	30/52 (57.8%)	52/80 (65.0%)	NS

*High quality embryos were embryos having a AEQS of 1.0 – 1.5.

As another part of this analysis we examined if a high level of estradiol (>4200 pg/ml) on the day of hCG had a deleterious effect on pregnancy rate when compared to a group having moderate estradiol levels of >3000 pg ml and <4200 pg/ml, that had embryo transfer on day 3. As shown in Table 9, the estradiol level and number of oocytes retrieved were higher in the >4200 pg/ml group, but there was no difference in either the CPR or LBR between groups.

**Table 9:** **Comparisons between patients having day 3 embryo transfer with moderate (>3000 < 4200 pg/ml) or high (>4200 pg/ml) estradiol levels on the day of hCG administration**

Variables	Estradiol Level		p value
	>3000 < 4200 pg/ml	>4200 pg/ml	
Number	63	22	
Estradiol level	3529 ± 303	4872 ± 619	< 0.0001
Oocytes collected	19.24 ± 8.12	25.0 ± 8.3	< 0.005
Number of 2PN embryos	8.35 ± 3.92	10.1 ± 4.15	< 0.08 NS
Number of embryos transferred	2.19 ± 0.40	2.09 ± 0.29	< 0.29 NS
AEQS	1.62 ± 0.66	1.51 ± 0.46	< 0.47 NS
CPR	37 (58.7%)	15 (68.2%)	NS
SAB	5 (15.6%)	3 (20.0%)	NS
LBR	32 (50.8%)	12 (54.5%)	NS

## Discussion

This study examined the relationship between estradiol level and other IVF cycle response parameters in relation to pregnancy, with a focus on live births after controlling for embryo quality. In high responders there is usually an increased number of eggs retrieved resulting in more embryos and therefore, better embryo selection, leading to more patients undergoing transfer on day 5. In the case of poor responders the opposite is true as was demonstrated in the low estradiol group in Table 2. Here, there were fewer oocytes in spite of a higher dosage of FSH, which also resulted in fewer 2PN embryos and ultimately poorer embryo selection at the time of transfer.

In our experience, patients having embryo transfer on day 3 can attain high pregnancy rates, but only if their embryo quality is not compromised. Therefore, we evaluated a population of patients undergoing their initial IVF cycle to determine the relationship between serum estradiol level and IVF outcomes. We next evaluated a subgroup that included patients having only good or fair quality embryos transferred to study the influence of embryo quality on pregnancy rate and subsequent live birth outcome. Although our initial categorical analysis showed that in an unselected population of first time IVF patients, estradiol level was significantly related to better pregnancy rates (Table 2), in patients having only good or fair quality embryos transferred, (to control for embryo quality influence), estradiol level was no longer related to pregnancy outcome (Table 3). Patients in the lowest estradiol group required the greatest amount of FSH, suggesting a significant component of this group was made up of poor responders. Since our primary interest was in understanding the relationship between live birth and elevated estradiol, we conducted a univariate analysis on the entire population to evaluate the association of IVF response variables with LB outcome. This analysis showed that patient age, estradiol level, FSH dose, number of MII oocytes, total number of 2PN embryos, AEQS and day of transfer (day 3 or day 5) were all related to LB rate. After multiple regression to adjust for confounders and to test for interactions, the only main effects that significantly affected LBR were, AEQS (P < 0.001), the transfer of two embryos (P < 0.01) and the patient’s age (P < 0.03). The level of estradiol had no effect on LB rate (P = 0.308). We also found significant two-way interactions between transfer on day 5 and patient age (P < 0.01) as well as transfer on day 5 and FSH dose (P < 0.04). We interpret these interactions to mean that patients achieving a LB after transfer on day 5 were younger, and also required less FSH.

Some studies [20, 21] have reported that estradiol was elevated in non-pregnant patients compared to pregnant patients suggesting that an elevated estradiol level may be associated with not achieving pregnancy, although in one study [20] this was seen in patients having a day 3 transfer but not on day 5. To examine this question, we partitioned patients on the basis of LB status by day of transfer. Results from our study (Tables 6 and 7) and another study [17] do not support this finding since estradiol was either higher or the same in LB patients compared to NLB patients.

Others [17, 20, 22] have proposed that elevated estradiol may have a negative influence on pregnancy but only when embryo transfer is performed on day 3, arguing that a supra-physiological estradiol environment may cause embryo-endometrial asynchrony which is alleviated if transfer is delayed until day 5. To address this question, we evaluated a subset of patients that had an estradiol level of > 3000 pg/ml on the day of hCG taken from the entire population (described in Table 2), which compares IVF cycle response variables for patients that had embryo transfer on day 3 or day 5. We chose to use the same estradiol cutoff range (>3000 pg/ml) used in a recent publication [20]. Our results (Table 8) show that in contrast to this report, we found no statistical difference in CPR or LBR in patients undergoing embryo transfer on day 3 or day 5 although CPR and LBR were slightly higher for patients having transfer on day 5. The study of Elgindy et al. [20] was a randomized controlled trial that had strict exclusion criteria (patient age <35 with regular cycles and a minimum of 4 good quality embryos on day 3 of culture). By design, the estradiol level, number of oocytes retrieved, number of 2PN embryos and number of good quality embryos were all similar for the day 3 and day 5 transfer groups. Although ours was a retrospective cohort study we believe it is more reflective of a typical population of patients undergoing an initial IVF cycle. In contrast to the referenced study [20] the day of embryo transfer in our study was decided based on the number and quality of embryos, an approach similar to that used in an earlier study [22]. Therefore some bias exists which placed more patients having numerous high quality embryos in the group having embryo transfer on day 5. In our study estradiol levels were not different between patients having transfer on day 3 or day 5 but there were a significantly greater number of oocytes retrieved and 2PN embryos with fewer embryos being transferred on day 5. Of the patients used in our study, 17% were diagnosed as having PCOS using the Rotterdam criteria [24] and therefore probably constituted a significant number of high responding patients. These differences may at least partially explain the divergent conclusions drawn from the two studies. To further examine if patients having an elevated level of estradiol on day 3 had a different pregnancy rate compared to patients having a moderate level of estradiol undergoing embryo transfer on day 3, we performed an additional subgroup analysis. Our high estradiol group had a level of >4200 pg/ml and the moderate estradiol group had a level >3000 and < 4200 pg/ml, which are the same ranges used by Elgindy et al. [20]. Our results, shown in Table 9, demonstrate that the average estradiol level and number of oocytes collected were higher in the high estradiol group but there was no significant difference in CPR or LBR when compared to the moderate estradiol group, which is a conclusion not supported in the previous referenced study [20]. Understanding and managing the high responding patient is a challenge to physicians in the clinical setting of IVF. There is an increased chance of patients developing ovarian hyper-stimulation syndrome (OHSS), which in our study was low, with only four patients out of 489 (0.08%) exhibiting moderate/severe OHSS that required paracentesis, all of which were in the moderate estradiol group (2000-4000 pg/ml) described in Table 2 and none of which had any long term adverse effects. Aside from the possibility of OHSS, much has been written about supra-physiological levels of estradiol and progesterone potentially causing endometrial-embryo asynchrony which could negatively impact pregnancy rates.

Several reports have shown that in high responding patients there is a concomitant increase in estradiol, progesterone and the number of retrieved oocytes [25–27] which may have a physiological linkage. In the case of high responders, the relationship between elevated levels of estradiol and progesterone was recently examined. Wu et al. [18] found that elevated levels of estradiol were not detrimental to live birth rate when progesterone levels were < 1.05 ng/ml whereas; when progesterone levels exceeded this threshold LB rates were compromised. Another study [27] examined this same relationship except the focus was on evaluating different progesterone thresholds. Their conclusion was that high progesterone correlated with high estradiol levels and that in high responders’ progesterone levels did not show any clinical impact, although a slight compromise was observed when a threshold of 1.8 ng/ml was exceeded. This study confirmed earlier work [28] which had shown that progesterone levels >1.5 ng/ml on the day of hCG reduced the chance of pregnancy in low and normal responders but not in high responders. On the basis of our results and the results of others, high responding patients are different than poor or normally responding patients regarding pregnancy outcomes and therefore this category of patients should be evaluated aside from the general population of patients. For this reason more large scale studies need to be performed that will elucidate if high responding patients are subject to hormone thresholds that if exceeded, could impair live birth rates. A limitation of our study was that progesterone levels on the day of hCG were not included, but unfortunately these values were not available. It is also important to point out that it is generally accepted that the supra-physiological hormone environment found in high responding patients does not have a negative effect on embryo quality or developmental potential. This has been borne out by studies in which embryos exposed to this environment and transferred to embryo recipients [11] or cryopreserved and transferred in a subsequent cycle [6] had no adverse effect on pregnancy rates. Results from our study indicate that, at least in the case of elevated levels of estradiol, no adverse effect on live birth rate is seen, regardless if embryo transfer was conducted on day 3 or day 5. Rather, our results show that the quality of transferred embryos and patient age are the primary determinants of having a live birth.

## Conclusions

We believe that our study has demonstrated that after controlling for embryo quality, an elevated level of estradiol on the day of hCG is neither directly detrimental nor advantageous to having a live birth. Our study shows that average embryo quality at transfer is the primary determinant in establishing a LB while patient age and the transfer of two embryos are also important. Significant interactions showed that there is interplay occurring between embryo transfer on day 5 and a lower dose requirement of FSH and younger patient age, both of which appear to contribute to an increased LB rate. An elevated level of estradiol should be viewed in the context of being part of the profile of a “high responder” requiring less FSH, more oocytes retrieved, more mature oocytes and more 2PN embryos. Yet, it is interesting that all of these response variables were found to not impact LB rate. On the basis of comparable live birth rates, our data also suggest that embryos of high quality appear to tolerate an arguably sub-optimal uterine environment that has been exposed to supra-physiological levels of estradiol.

While our study has demonstrated that elevated estradiol has no effect on live birth rate, in the light of some recent studies [29–31] showing that elevated estradiol may interfere with some aspects of placentation which in turn can contribute to an increased incidence of preeclampsia and small for gestational age (SGA) offspring, it is imperative that larger prospective studies be conducted to determine if this hormone environment is responsible for these effects. If the initial findings are substantiated perhaps it is time to seek a more comprehensive definition of IVF success that encompasses fetal and neonatal well-being and to look beyond live birth as the singular metric by which IVF success is defined.

## Abbreviations

2PN: Embryos with two pronuclei
ANOVA: Analysis of variance
AEQS: Average embryo quality score
BCP: Biochemical pregnancy
CP: Clinical pregnancy
CPR: Clinical pregnancy rate
FSH: Follicle stimulating hormone
GnRH: Gonadotropin releasing hormone
GPS: Global protein supplement
hCG: Human choronic gonadotropin
hMG: Human menopausal gonadotropin
HSA: Human serum albumen
HTF: Human tubal fluid
ICM: Inner cell mass
ICSI: Intracellular sperm injection
IM: Intramuscular
IVF: In Vitro Fertilization
LB: Live birth
LBR: Live birth rate
MII: Metaphase II
mHTF: Modified (HEPES) human tubal fluid
NLB: Non-live birth
TE: Trophectoderm.
